# Small world in the real world: Long distance dispersal governs epidemic dynamics in agricultural landscapes

**DOI:** 10.1016/j.epidem.2020.100384

**Published:** 2020-03

**Authors:** Giovanni Strona, Claudio Castellano, Simone Fattorini, Luigi Ponti, Andrew Paul Gutierrez, Pieter S.A. Beck

**Affiliations:** a Research Centre for Ecological Change, University of Helsinki, P.O. Box 4, FI-00014, Finland; b Istituto dei Sistemi Complessi (ISC-CNR), Via dei Taurini 19, 00185 Rome, Italy; c Department of Life, Health and Environmental Sciences, University of L’Aquila, Via Vetoio, Coppito, 67010, L’Aquila, Italy; d Agenzia nazionale per le nuove tecnologie, l’Energia e lo sviluppo economico sostenibile (ENEA), Centro Ricerche Casaccia, Via Anguillarese 301, 00123, Roma, Italy; e Center for the Analysis of Sustainable Agricultural Systems (CASAS Global), 37 Arlington Ave., Kensington, CA, 94707-1035, USA; f Division of Ecosystem Science, College of Natural Resources, University of California, Berkeley, CA, 94720-3114, USA; g European Commission, Joint Research Centre (JRC), Ispra, Italy

**Keywords:** Monoculture, Olive, Outbreak, Quarantine, Vector-borne disease, *Xylella fastidiosa*

## Abstract

Outbreaks of a plant disease in a landscape can be meaningfully modelled using networks with nodes representing individual crop-fields, and edges representing potential infection pathways between them. Their spatial structure, which resembles that of a regular lattice, makes such networks fairly robust against epidemics. Yet, it is well-known how the addition of a few shortcuts can turn robust regular lattices into vulnerable ‘small world’ networks. Although the relevance of this phenomenon has been shown theoretically for networks with nodes corresponding to individual host plants, its real-world implications at a larger scale (i.e. in networks with nodes representing crop fields or other plantations) remain elusive. Focusing on realistic spatial networks connecting olive orchards in Andalusia (Southern Spain), the world’s leading olive producer, we show how even very small probabilities of long distance dispersal of infectious vectors result in a small-world effect that dramatically exacerbates a hypothetical outbreak of a disease targeting olive trees (loosely modelled on known epidemiological information on the bacterium *Xylella fastidiosa*, an important emerging threat for European agriculture). More specifically, we found that the probability of long distance vector dispersal has a disproportionately larger effect on epidemic dynamics compared to pathogen’s intrinsic infectivity, increasing total infected area by up to one order of magnitude (in the absence of quarantine). Furthermore, even a very small probability of long distance dispersal increased the effort needed to halt a hypothetical outbreak through quarantine by about 50% in respect to scenarios modelling local/short distance pathogen’s dispersal only. This highlights how identifying (and disrupting) long distance dispersal processes may be more efficacious to contain a plant disease epidemic than surveillance and intervention concentrated on local scale transmission processes.

## Highlights

• Pathogens’ long distance dispersal might drive epidemics in agricultural landscapes.
• We explored the relevance of long distance dispersal using network analysis.
• We simulated insect-born outbreaks of *Xylella fastidiosa* on olives in Andalusia.
• Probability of pathogen’s long distance dispersal dramatically affected outbreaks.
• Limiting pathogen’s dispersal might be more effective than local control strategies.

## 1 Introduction

Disease spread within an agricultural field can be realistically modelled using a lattice where nodes (plants) are regularly spaced (Newman et al., 2002). Similarly, to model the spread of a disease across a monoculture agricultural landscape, we can use a network where nodes represent individual crop fields, with links connecting nodes depending on explicit spatial rules. Such connections represent hypothetical pathways for disease transmission between fields (Strona et al., 2017).

In a simplified scenario where a pathogen’s maximum dispersal ability is quite limited, and comparable to the average distance between a crop field and its closest neighbor across the region, the resulting network will have structural properties similar to those of a random geometric graph (Penrose, 2003). Epidemic dynamics in such a network are relatively simple. In a typical Susceptible-Infected-Removed model (Pastor-Satorras et al., 2015), starting from the initial seed infection, the removed nodes (i.e. the plants hit by the epidemics and recovered or dead, and thus no longer infectious) approximately form a disk around the seed, its radius growing over time. Infected nodes sit at the edge of the disk, forming an approximate ring. This makes the progression of the spread not only easy to model, but also easy to manage, for example by making all susceptible nodes surrounding an infected one immune to the pathogen. Conveniently, the number of nodes to be immunized grows only linearly with the number of infected nodes, due to the two-dimensional nature of the spatial network, in contrast with the more complex, and less manageable dynamics observed in non-spatial networks (Barthélemy et al., 2005; Strona and Castellano, 2018).

A common measure by which nodes are practically immunized in agriculture and forestry is the eradication of infected plants and all susceptible hosts within a certain radius from them (Parnell et al., 2009), and/or the establishment of control belts where all hosts are preventively monitored, treated, and/or removed (Gordh and McKirdy, 2014). This strategy to manage epidemics in vegetation, often also referred to as quarantine, is challenging because it requires rapid and complete implementation of measures that can impact multiple elements of society (Gordh and McKirdy, 2014). Thus, this approach, particularly in tree crops and forests, is often unsuccessful (Gottwald and Irey, 2007; Irey et al., 2006; Santini et al., 2013) and raises questions about the validity of the assumptions behind the spatial network models in actual plant disease epidemics.

The simplified scenario above assumes that the infection can be transmitted only to and from nearby nodes. However, both when pathogens are transmitted directly from one plant to another, and moved around by vectors, it is possible that the ‘true’ network includes additional, ‘temporary’ links. Such links can be formed by occasional movements of infectious agents or material at distances far beyond the natural dispersal ability of either the pathogen or the vector, resulting, for example, from meteorological events (e.g. strong winds) or human activity. Indeed, it is well-known that for small insects of phytopathological relevance, dispersal by wind constitutes a major impediment to the success of large-scale control strategies (Arthur and Bauer, 1981). Moreover, many small insects, such as aphids, deliberately exploit wind currents as a means for long distance dispersal (Compton, 2002; Gutierrez et al., 1974), possibly making dispersal by wind a systematic – albeit largely unpredictable – threat. Thus, in reality, a range of intermediate scenarios present themselves for plant disease epidemics where limited pathogen/vector mobility confers a strong spatial structure on the network, that, however, can be altered to different degrees by more or less frequent infections over longer distances.

Introducing the possibility of long distance dispersal in a network, by adding some links between geographically distant nodes, can substantially alter network structure. In particular, it is expected to progressively transform a spatial network into a small-world network (Watts and Strogatz, 1998), i.e., a network where any node can be reached from any other one by means of only a few hops along the network links. Since epidemic dynamics are strongly driven by network structure, long distance connections in a network can change not only epidemics, but also the viability of strategies to halt, slow, or control them (Newman et al., 2002). For example, previous theoretical work has shown how the abundance and the dispersal ability of vectors has important consequences for epidemic dynamics and intervention strategies: control measures become very costly even when vectors spend most of their time locally but are capable, occasionally, of long jumps (Dybiec et al., 2009).

An underestimation by decision makers of such small-world effects might have contributed to real world examples where typical containment strategies have struggled to halt epidemics in apparently static spatial networks (de la Fuente et al., 2018). Hence, a better understanding of this phenomenon could offer important insights for a more informed management. In particular, it may provide solid theoretical bases to assess the relative importance of broad monitoring campaigns aimed at limiting the probability that random dispersal events could disrupt the network’s intrinsic spatial pattern. Here, we investigate how increasing the probability of long distance dispersal events could transform epidemic dynamics in a regular spatial network, taking as a case study, the potential spread of the bacterium *Xylella fastidiosa* in olive trees in Andalusia, Spain, the world’s most productive region for olive oil.

The pathogen *X. fastidiosa*, an important pest of citrus and grapevine in the Americas, has recently become a major threat to Mediterranean agriculture. In 2013, the pathogen was detected in Southern Italy in olive trees (a new host at the time), and the damage caused by its rapid spread caused a phytosanitary emergency (Martelli et al., 2016; Saponari et al., 2013, 2017). Previous work has shown that olive-dominated landscapes in Southern Italy are prone to *X. fastidiosa* establishment (Strona et al., 2017) which implies that other regions where olive trees constitute an important resource should remain vigilant. Most prominent among these is Spain, which, with an estimated production of 1.55 × 10^6^ t of olive oil for the crop year 2018–2019 (from October 1 to September 31), accounts for more than 50% of the world’s production (3.064 × 10^6^ t) (International Olive Council, 2018). Recent discoveries of the pathogen on the Balearic Islands, in the nearby Spanish region of Valencia and, more recently, in Andalusia (Junta de Andalucía, 2018) highlight the risk of a *X. fastidiosa* epidemic in Andalusia, the world’s foremost olive oil producing region (Galán et al., 2008), hosting more than 75% of Spanish olive oil farms (European Commission, 2012), even if the Spanish strains are different from the Apulian one (hence possibly obeying different epidemiological rules). Here we provide strong numerical evidence that, if the risk materializes, long distance vector dispersal events will be the main driver of epidemic dynamics.

## 2 Methods

We created a spatial network based on geographical proximity between Andalusian olive orchards. Orchard distribution data were obtained (in the form of 206,599 geospatial polygons representing individual orchards) from the land use and vegetation cover map of the Environmental Information Network of Andalusia (Spanish National Geographic Institute, 2013). We then created a network connecting all pairs of orchards having *D_ij_* smaller than 500 m, with *D_ij_* being the shortest linear distance between the *i*-th and *j*-th polygons (measured from their closest sides).

We then modelled the spread of the disease across the network using a Susceptible – Infected – Removed (*SIR*) model (Pastor-Satorras et al., 2015). Nodes can be either healthy but susceptible to contracting the infection (state *S*), infected and able to transmit the disease to their neighbors (state *I*), or removed, recovered or dead, and thus no longer participating in the process (state *R*). The model assumes that, at each step, all infected nodes transmit the infection to each of their neighbors in the *S* state with probability *q* and then switch to state *R* with probability *r*.

We focused on a vector borne disease, hence assuming a two-step mechanism for the pathogen’s transmission from one node to another, with the first step being the movement of the vector from an infected node to a susceptible one, and the second step being the actual infection of hosts in the susceptible node/orchard. For this, we had to first identify the number of vectors moving from one orchard to another one. The list of potential insect vectors for *X. fastidiosa* as a pathogen of olive trees is very long, with most sap-feeding insects (particularly spittlebugs) being candidates (Cornara et al., 2014, 2017; Martelli et al., 2016). Among those, the meadow spittlebug *Philaenus spumarius* has been identified as the most important vector in Italy (Saponari et al., 2014; Cornara et al., 2014, 2017).

Populations of *P. spumarius* can reach very high densities in suitable conditions. However, in Europe, the maximum recorded density of nymphs does not exceed 1000 individuals/m^2^, and is expected to be considerably lower in managed crops (Cornara et al., 2018). A recent survey conducted in various regions of Spain also suggests that *Philaenus spumarius* is present at moderate densities (Morente et al., 2018). In a recent study conducted in Italy, Ben Moussa et al. (2016) provide year-round monthly variation in various sap-feeding insects’ abundances, including *P. spumarius*. By transforming their estimates into relative abundances, and assuming an arbitrary (yet reasonable) annual peak of 20 individuals/m^2^, we obtained a placeholder for monthly average *P. spumarius* density (particularly *PsD_x_* = 0.54, 0.0, 0.06, 3.36, 4.3, 7.4, 10.44, 13.6, 20.0, 3.44, 0.68, 0.48 adults/m^2^ from January to December). Note that the density peak in Spain, according to Morente et al., 2018, seems to occur earlier than in Italy. However, the shape of the yearly trend is qualitatively consistent with the one identified by Ben Moussa et al. (2016).

Then, we conservatively assumed that only vectors close to the boundary of the target orchard (i.e. within ∼10 m from the boundary) can leave ‘their own’ orchard and move to other ones. Thus, we quantified the number of vectors attempting dispersal from their own orchard to other ones at a given time step (i.e. month) as:

(1) $$Vn=\frac{\left(10m\times P_{i}\times {PsD}_{x}\right)}{n_{i}}$$

With *P_i_* being the perimeter of the *i*-th node/orchard of origin (i.e. from which a vector moves), *PsD_x_* being the monthly abundance of vectors per square meters at month *x*, 10 being the width of the boundary zone from which we expect vectors to move to other nodes/orchards connected in the network to the target one, and *n_i_* being the number of nodes connected to node *i*. We then computed the number of vectors involved, at each step, in long distance dispersal events as:

(2) $${Vn}_{LDD}=Vn\times \delta$$

We explored 11 scenarios spanning one order of magnitude, with δ = [0, 0.00005, 0.00010, 0.00015, …, 0.0005] being the probability of long distance dispersal (LDD). We then computed the number of vectors involved in local dispersal as:

(3) $${Vn}_{loc}=Vn-{Vn}_{LDD}$$

We attributed to each link in the network between the *i*-th and *j*-th node a weight corresponding to the probability of a vector (either infectious or not) moving further than *D_ij_* over the time interval in question, assuming that the distance moved produces an exponential spread process (as in White et al., 2017).We modelled this probability, ideally representing the dispersal kernel for *Xylella fastidiosa* vectors, to be:

(4) wij=ifDij≤500m→exp-Dij/100mifDij>500m→0

That is, we assumed that, on average, vectors moved 100 m in one month, but no further than 500 m. Such estimate of vector dispersal ability, although higher than the choice of White et al., 2017, who used the same kernel to model yearly movements (even if without the 500 m constraint, and implicitly assuming that vectors are active only during summer season), is still conservative, considering that experimental work has shown that *P. spumarius* is actually capable of movements exceeding 100 m *per day* (Weaver and King, 1954).

Following White et al. (2017), we modelled the progression of the infection within a single node in the network through time by identifying the proportion of infected hosts in a given orchard (within node infection prevalence) as a Gompertz growth with a maximum proportion of 1 (see Fig. S1A):

(5) $$N_{i}\left(t\right)=exp\left[-14.069\times exp\left(\frac{-0.25}{Months}\times t\right)\right]$$

The parameter *t* indicates time (in months) since the first infection in the target node. Note that White et al. (2017) have used a time resolution of 1 year, and therefore multiplied *t* by -3 instead of by -0.25. We defined the probability of infection transmission from an infected node *i* to a susceptible node *j* as:

(6) $$q_{ij}={1-\left[1-\pi \times {Xf}_{j}\right]}^{{Vn}_{loc}\times N_{i}\left(t\right)\times \overset{´}{w_{ij}}}$$

Here, π is a factor expressing the overall infectivity of the pathogen, *Xf_j_* is the local climatic favorability to *Xylella fastidiosa* (see below), $\overset{´}{w_{ij}}$ is the normalized probability of a vector to move from node *i* to node *j* (that is $w_{ij}/\sum_{j}w_{ij}$), *N_i_* is the proportion of infected hosts in the source node *i* at time *t*, and *Vn_loc_* is the number of vectors moving during the monthly simulation step. In our simulations, we explored 9 different scenarios with π = [0.1, 0.2, …, 0.9]. The probability of successful transmission, *q_ij_*, combines the probability of a vector to move from one orchard to a neighboring one (*w_ij_*), the probability for that vector to be infectious at a given time [for which we used, as a proxy, the proportion of infected hosts at that moment, *N_i_(t)*], and the probability that the vector will infect susceptible hosts in the target node, given, in turn, by the overall pathogen’s infectivity, π, kept stable throughout a given simulation, combined with the climatic suitability to *Xylella* for the target node (*Xf_j_*).

For the last parameter, *Xf_j_*, we used the normalized growth rate index for *Xylella fastidiosa* (Gutierrez et al., 2011). This was computed using a normalized symmetrical scalar function that captures the thermal limits and the optimum at the midpoint of the favorable temperature range (dashed line in Fig. 7a from Gutierrez et al., 2011), with thermal limits identified by modeling the in vitro growth rate of *Xylella fastidiosa* on temperature (solid line, Fig. 7a from Gutierrez et al., 2011) based on data reported by Feil and Purcell (2001). We computed the index on a regular geographical grid of 0.25˚ × 0.25˚ covering both Spain and Portugal (including their islands). The annual sum of the daily favorability index *Xf* was computed for the period 1 January 1981 to 31 December 2010 using daily maximum and minimum temperature from the freely available AgCFSR weather data set (Ruane et al., 2015) at each grid location. The annual sums of the daily favorability index values were averaged across the 30-year period at each location and then the averages were normalized using the maximum value across all locations. We decided to normalize the index over such a large area (rather than over Andalusia only) in order to capture a meaningful range of variability in the index values, and avoid artifactually high variability in the Andalusian region following the normalization.

We then quantified the probability of a vector to successfully disperse from node *i* to another random node *j* in the network (either infected or not) as:

(7) qLDDij=ifdij⩽rd→1-1-π×XfjVnLDD×Nitifdij>rd→0

where $d_{ij}$ is the distance between the orchards *i* and *j*, and *rd* is the value of a random number sampled from a folded normal distribution with mean 50 km and standard deviation 150 km (see Fig. S2)with a new *rd* value sampled for each *ij* pair of nodes).

We set the probability of a node to be removed from the network, that is to pass from the infected (*I*) status, to the removed (*R*) one as:

(8) $$r\left(t\right)=exp\left[-14.069\times exp\left(\frac{-1}{Months}\times t\right)\right]$$

As in the previous equations, *t* indicates the number of months since the node had been infected. It is evident that we modelled the probability of recovery using the same model we used for the progression of the infection within an orchard, but assuming a much faster growing curve (Fig. S1B). In doing this, we tried to capture the two ideas (which are clearly an oversimplification of reality) that the higher the prevalence of infection within an orchard, the higher the likelihood for the infection to be detected and the orchard to be ‘treated’; and that, in a scenario of prompt intervention, this should happen well before all hosts are infected (even though, in the case of *Xylella* and in many other real world situations of agricultural diseases, this scenario might be overly optimistic due to the potential time lag between the moment of the infection, and the appearance of detectable symptoms)

We ran 1000 epidemic simulations for a maximum time of 5 years (i.e. 60 monthly steps *t*) for each one of the 110 possible combinations of *π* and *δ*, and each time starting from a single, randomly selected, infected node. During each simulation, we kept track, at each monthly step, of the relative proportion of nodes in the different epidemic compartments (*S*, *I*, *R*) and of the total infected area, given by the cumulative sum of areas of infected nodes. Additionally, we evaluated the efficacy of ‘optimal’ quarantine, which is here defined as the removal of each healthy node directly connected to one or more infected nodes (Strona and Castellano, 2018). This definition is based on the assumption that infection events are instantly known, and that the implementation of quarantine is instantaneous as well. In other words, we do not consider common real world situations where, due to some delay in intervention, a node is being quarantined *after* becoming infectious. In those cases, quarantining immediate neighbours only will not guarantee success. Thus, we kept track of the proportion of nodes that should have been, at a given step, hypothetically quarantined to halt the epidemic. However, we ran simulations as if quarantine was never applied, letting the infection dynamics play out freely for all the 60 steps (or until no infected nodes remained in the network when this happened in less than 5 years from the first infection). We then evaluated how the dynamics and the outcome of a simulated spread (measured as the proportion of nodes ultimately reached by the infection), depended on *π* and *δ*.

To evaluate the effect of π and δ on the effort ideally required by the implementation of quarantine, we performed linear regressions (across the 1000 epidemic simulations we ran for each combination of π and δ) between the number of newly infected nodes at each temporal step and the corresponding number of nodes that one should have removed from the network to immediately halt the outbreak (those correspond to all nodes neighboring the infected node, and in the susceptible state, *S*). Then we took the slopes of the regression lines (ideally representing the the change in the average number of nodes that need to be quarantined per infected node) as a measure of quarantine effort.

## 3 Results

**Fig. 1:**
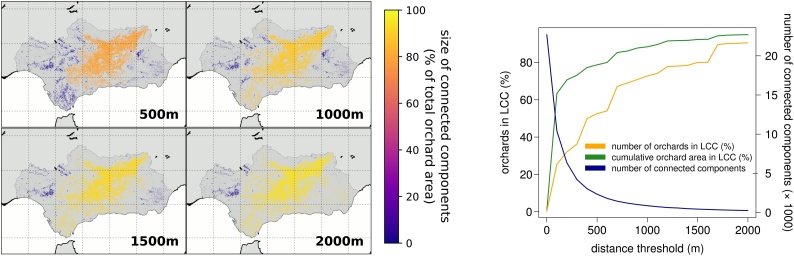
Most Andalusian olive orchards are connected even for a pathogen with limited dispersal abilities. We investigated how the connectivity between olive orchards in Andalusia varies for increasing threshold distances that represent the maximum dispersal range of a hypothetical infectious agent. Panel **A** shows the distribution and size of connected components (i.e. individual clusters of orchards in which each member, i.e. orchard, has at least one other orchard closer than the selected threshold distance) for increasing threshold distances (reported, in meters, at the bottom right of each map). Panel **B** reports the variation in the size of the largest connected component (LCC), expressed as the percentage of both the number of orchards (orange line) and as the combined orchard area (green line), and as the total number of components (including isolated orchards) for increasing threshold distances (blue line). (For interpretation of the references to colour in this figure legend, the reader is referred to the web version of this article.)

The average distance between an orchard and its closest neighbor in the region of Andalusia is extremely small (28 m ± 128 S.D., with a maximum of 6403 m, computed over 206,599 orchards) and can be easily covered by active dispersal of known insect vectors of *Xylella fastidiosa* (Cornara et al., 2018, 2019). As a consequence, a network built by connecting all orchard pairs that are closer than a threshold distance chosen based on the maximum dispersal ability of an infectious agent (Strona et al., 2017), will encompass most of the olive orchards in the region even when such distance is short. For a threshold distance of 500 m (the same we used to build our network), 79 % of olive orchards are connected into a single giant component (Fig. 1).

**Fig. 2:**
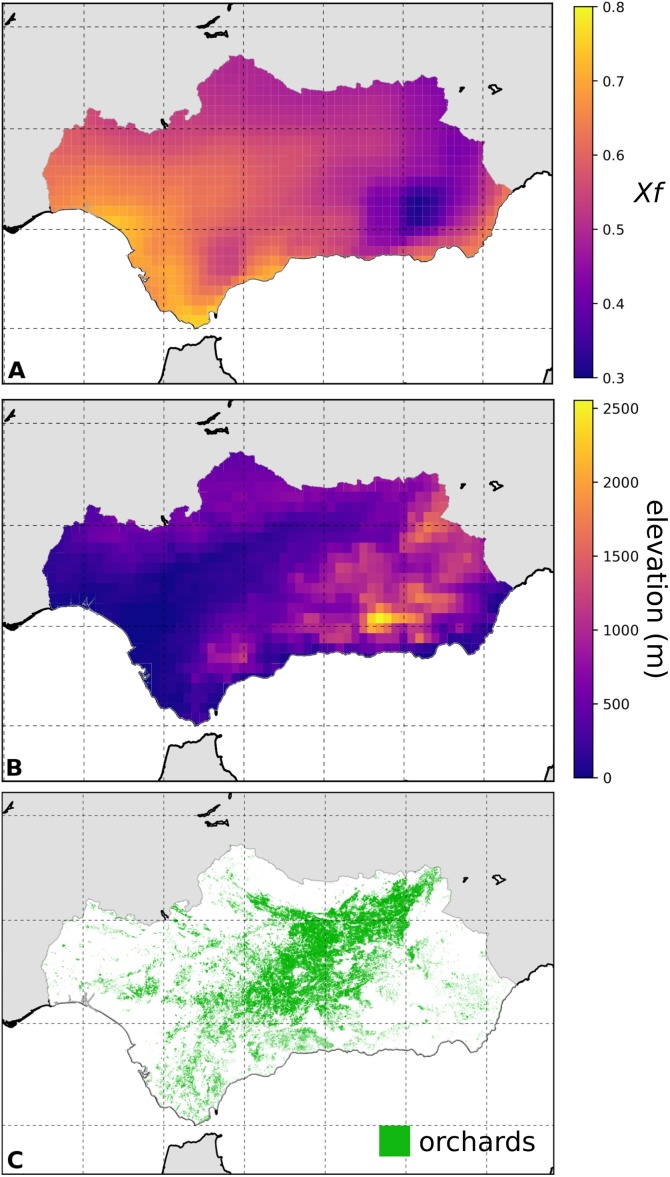
Relative climatic suitability in Andalusia for *Xylella fastidiosa*. The index of suitability (*Xf*) was computed according to Gutierrez et al.^25^. *Xf* is a normalized growth index computed by feeding daily maximum and minimum temperatures (retrieved from https://data.giss.nasa.gov/impacts/agmipcf/) to a normalized symmetrical scalar function that captures the thermal limits and the optimum at the midpoint of the favorable range for *Xylella*. The index was calculated on a geographical grid of 0.25˚ × 0.25 ˚ for both Spain and Portugal (including islands); the annual cumulative sums of *Xf* for years 1981–2010 were then averaged in each grid cell across the 30-year period and normalized across the cells. The map shown here of *Xf* in Andalusia was obtained by triangular interpolation of the index to 0.1˚ resolution (A). *Xf* was used in the epidemic (SIR) simulations to weight the overall pathogen’s infectivity (π) so that the probability that, at a given simulation step, an infected orchard transmitted the infection to a neighboring healthy one was set to π multiplied by *Xf* of the healthy orchard. To help interpretation, we also report a map showing elevation in the region (in meters, interpolated across the same grid used for the *Xf* index to ease visual comparison) (B), and a map showing the distribution of olive orchards in the region (C). (For interpretation of the references to colour in this figure legend, the reader is referred to the web version of this article.)

Climatic suitability for *Xylella fastidiosa* varies considerably across Andalusia (Fig. 2A). The pathogen is adapted to hot weather, with thermal limits of 12.5–32.25 °C and an optimum at 22.38 °C, as illustrated by its distribution in California, where both the vector and the pathogen are primarily found in the hot desert regions (Gutierrez et al., 2011). In northern California, the colder conditions are more limiting to the pathogen (Feil and Purcell, 2001; Purcell, 1980, 1997) than to its vector, which has slightly broader thermal tolerances. Given the wide geographic distribution of olive trees in Andalusia, and the hot-summer Mediterranean climate the region experiences, the areas of highest predicted favorability for *X. fastidiosa* are at lower elevations or in sectors where climate is moderated by the sea (Fig. 2B) and, coincidently, where most olive trees occur (Fig. 2C).

**Fig. 3:**
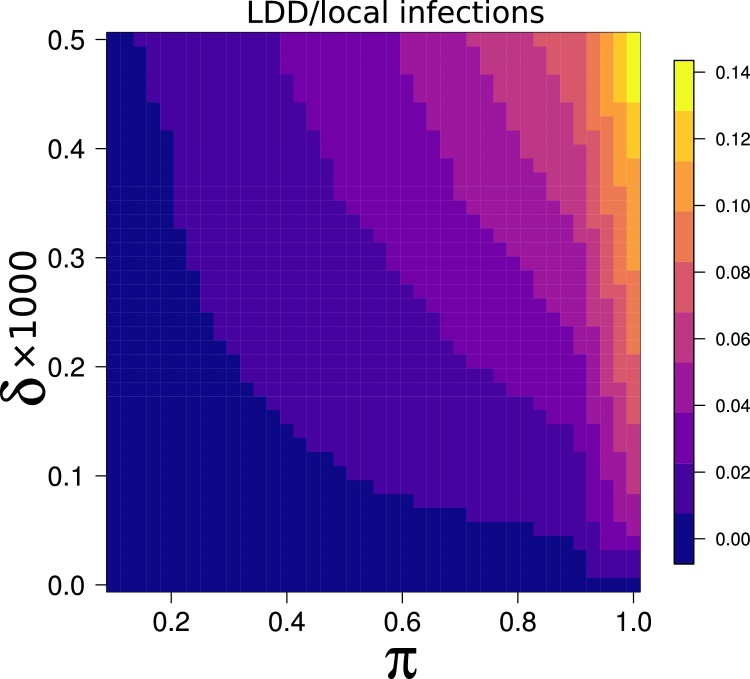
Average ratio between the number of successful vector long distance dispersal events (i.e. leading to new infections), LDD, and the number of local infections under different combinations of π and. δ.

There are many factors that can affect the frequency of long distance dispersal (LDD) events in a simulation besides the obvious effect of *δ* (see Methods). Therefore, we kept track of the actual number of successful long distance infections in a given simulation, and compared that with the corresponding number of local infections, to verify that long distance dispersal events were not unrealistically prevalent in a simulation. The selected range of *δ* values resulted in small proportions of successful long distance dispersal events (i.e. leading to a novel infection) compared to the total number of infections within an outbreak. Such percentages increased with both *δ* and π (Fig. 3). For low probability of LDD and low pathogen infectivity (δ = 0.00005 and π = 0.1), the percentage of successful long distance infections over the total of infection events was as low as 0.05% ± 0.4 (mean ± S.D.). For intermediate values (δ = 0.00025 and π = 0.5), the percentage increased to 1.7% ± 1.7, and reached 13.6% ± 8.4 in the worst case scenario of very high LDD probability and pathogen infectivity (δ = 0.0005 and π = 1).

**Fig. 4:**
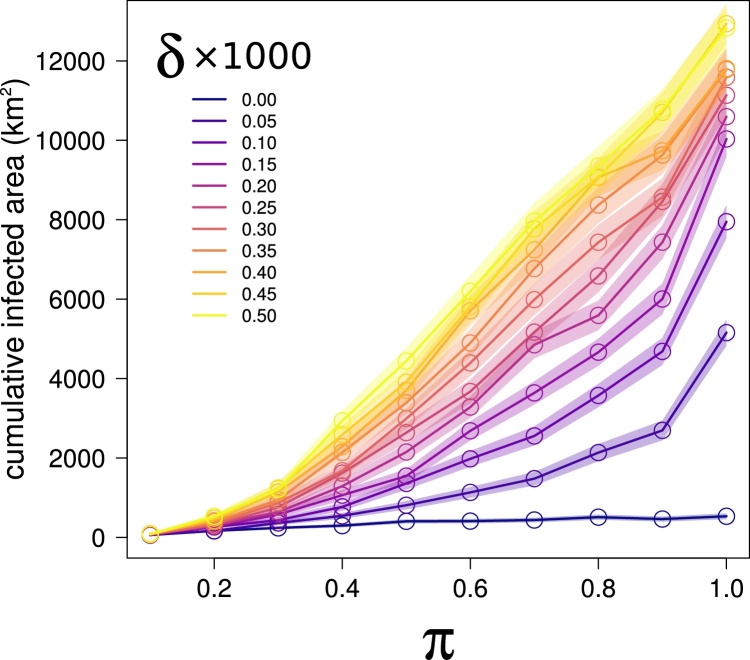
The total area of orchards reached by the infection after 5 years of simulations is strongly affected by δ, with the effect becoming more visible as π increases. We compared the total area encompassed by nodes reached by the infection at the end of an epidemic simulation with the overall infectivity (π) under different scenarios of long distance dispersal probability, δ. Each point represent the mean value of the 1000 simulations conducted for a given combination of π and. δ.

Nevertheless, even a small number of successful long distance dispersal events was enough to produce a small-world effect (Watts and Strogatz, 1998) which substantially altered epidemiological dynamics, and eventually resulted in much larger infected areas at the end of the simulations (Fig. 4). For small values of π (≤0.3), the total area reached by the infection remained low, with a slow contribution from *δ*. However, for higher values of π the differences in the outcome of epidemic simulations driven by δ became steadily larger, leading also to situations where the importance of long distance dispersal surpassed that of infectivity. For example, simulations with π = 0.4 and a high *δ* (e.g. = 0.0005) resulted in a much larger infected area than several other simulations with a much higher π, but lower *δ*. For π = 1, the average total infected area for simulations where long distance dispersal was not modelled (i.e. with *δ* = 0) was 9.7 times smaller than that obtained in simulations where very weak long distance dispersal was modelled (i.e. *δ* = 0.00005), and more than 24 times smaller than the average infected area obtained from simulations with the largest *δ* (0.0005).

**Fig. 5:**
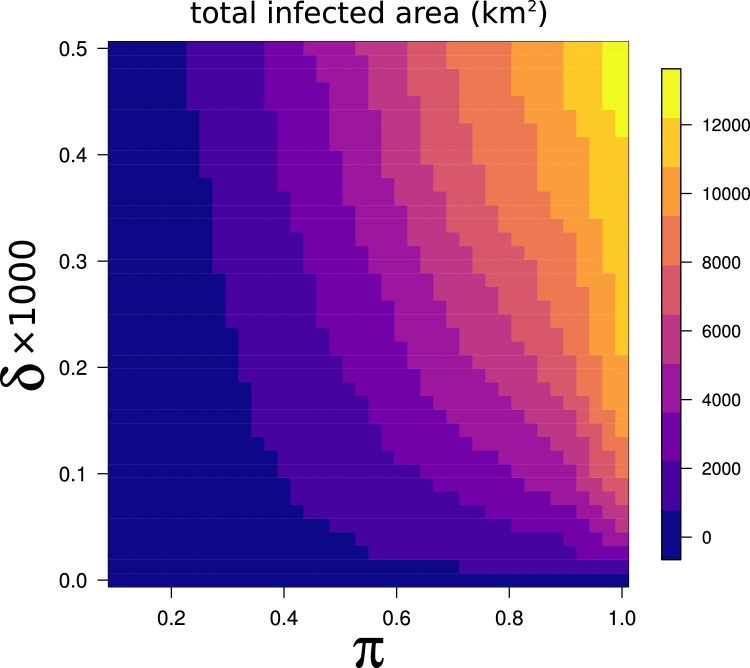
Different combinations of π and δ can lead to similar epidemic outcomes in terms of total infected area. The colors correspond to the average cumulative infected area after 5 years from the first infection interpolated at different values of π and. δ.

The largest infected areas were found in simulations with both high *π* and *δ*. Very high values of *π*, representing a very infectious pathogen, did not lead to large outbreaks when long distance dispersal events were rare. Furthermore, many different combinations of *π* and *δ* eventually resulted in very similar outcomes in terms of total infected area after 5 years (Fig. 5).

These results apply to hypothetical situations where the pathogen is left to spread unchallenged, providing a much-needed frame of reference to benchmark potential management actions (Strona and Castellano, 2018). In the case of diseases such as those caused by *Xylella*, these actions often include the removal of infected nodes (i.e. plants). In an ideal scenario of perfect intervention, this is done promptly, where ‘promptly’ means before infected nodes have any opportunity to infect other ones. In reality, however, we can expect some latency between the moment a node is infected, and the moment action is taken, meaning many infected nodes do have a chance to infect other nodes. The ideal scenario, which halts the infection immediately, is thus unlikely in real-world situations. Therefore, quarantine is usually implemented, meaning that also neighboring nodes are removed, to increase the odds of stopping an infectious disease from spreading.

The quarantine effort required to halt/slow down an ongoing epidemic clearly depends on the number of infected nodes at the time when intervention is applied, and on the amount of susceptible nodes directly linked to the infected ones, which are those at immediate risk of contagion, and requiring quarantine. In the case of an infection spreading only locally from one node to neighboring ones, the volume of nodes to be quarantined tends to remain low because many neighbors of infected nodes will be in the infected or removed status as the infection progresses. Conversely, long distance infections cause a disproportionate increase in the number of nodes requiring quarantine. To investigate the magnitude of this effect, we modelled the relationship between the number of infected nodes and the number of nodes to be quarantined for increasing values of δ and π. In all cases, the relationship between the number of infected nodes and the number of nodes to be quarantined throughout a given simulation was very strong and linear (with an average R^2^ of 0.84 ± 0.03; min = 0.76, max = 0.88).

**Fig. 6:**
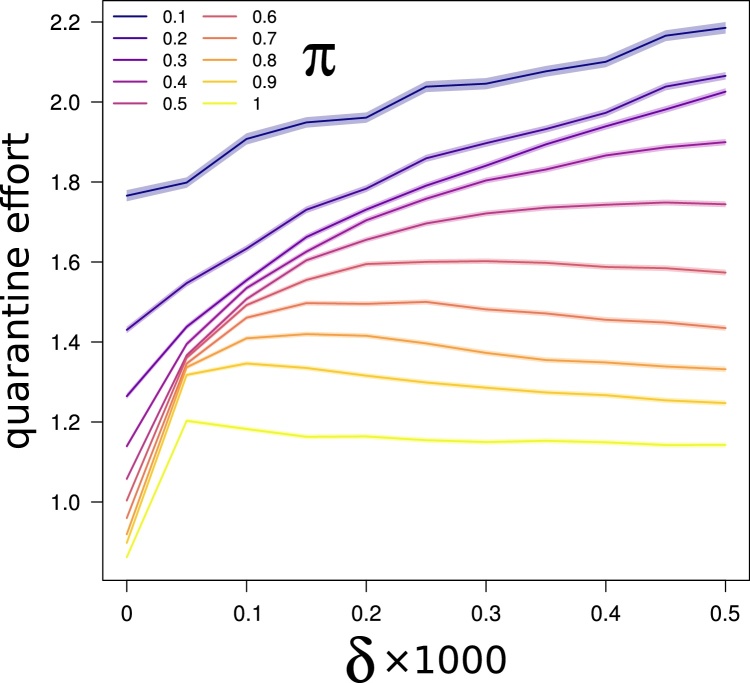
Long distance dispersal increases quarantine effort up to 1.6×. We performed linear regressions (across the 1000 epidemic simulations we run for each combination of π and δ) between the number of nodes infected at each temporal step and the corresponding number of nodes that one should have removed from the network to immediately halt the outbreak (those correspond to all nodes neighboring the infected node, and in the susceptible state, *S*). Then we took the slopes of the regression lines (ideally representing the average number of nodes that need to be quarantined per infected node) as a synthetic measure of quarantine effort. Finally we plotted these measures of quarantine effort (that is the slopes of the regression lines) vs. increasing values of δ. Shaded areas correspond to the 95% confidence intervals of regression lines’ slopes. Different lines in the plot correspond to different scenarios of overall pathogen infectivity (π). In all cases, the relationship between the number of infected nodes and the number of nodes to be quarantined throughout a given simulation resulted very strong and linear (with an average R^2^ of 0.84 ± 0.03).

For an intermediate scenario with π = 0.5, when δ = 0, that is in absence of long distance infections, on average 1.06 nodes needed to be quarantined for each infected node. However, this value immediately exceeded 1.4 (and remained above it) for any δ > 0, reaching a value of 1.74 for the largest considered value of δ (i.e. 0.0005; Fig. 6). For a very high pathogen infectivity (π = 0.5), the effect of even a very small probability of long distance dispersal (δ = 0.00005) is disproportionate, increasing the required quarantine effort by almost 30% compared to a scenario without long distance dispersal. For decreasing values of π, the effect of δ on quarantine effort is reduced, while therelative quarantine effort (number of nodes to be quarantined per infected node) increases. The latter result is partly explained by the fact that the probability of a node neighboring an infected one to be uninfected (and then target of quarantine) is positively affected by π at any step of the simulation. Thus, the number of nodes to be quarantined per infected node decreases for increasing values of π not as a consequence of a milder outbreak, but of a shrinking availability of susceptible nodes.

Furthermore, as shown above, the number of successful long distance infection events does not depend only on δ, as it is also non-linearly modulated by π (see Fig. 3). Thus, it is not surprising that the effect of δ on quarantine effort will be weaker for scenarios having low values of π, since those will be comparatively less impacted by LDD than scenarios having higher π.

## 4 Discussion

In principle, from a purely theoretical and modelling perspective, epidemic processes are relatively easily modelled and interpreted in networks with a spatial structure approaching a regular lattice that is a non-random regular network where each node is connected to all of its nearest neighbors. There, the front of a hypothetical outbreak moves from the initial seed infection as an approximate circle with its radius growing over time. Such an epidemic scenario would be also easily managed through a quarantine strategy that can immunize all susceptible nodes surrounding an infected one. The two-dimensional nature of the spatial network namely grows the number of nodes to be immunized only linearly with the number of infected nodes, in contrast with less manageable scenarios observed in other kinds of networks (Barthélemy et al., 2005; Strona and Castellano, 2018).

The fact that a regular lattice is a good approximation of the ideal network mapping short distance infection pathways between neighboring crop-fields in agricultural landscapes characterized by very low or no crop diversity (as in the case of olive orchards in Andalusia, or in Southern Italy, see Strona et al., 2017) might deceivingly depict an overoptimistic scenario in terms of epidemic risk and management effort.

Yet, our results reveal how subtle changes in network properties can drastically alter the dynamics of an infectious plant disease epidemic. Even if they are very rare, instances of long distance vector dispersal can profoundly disrupt such scenarios, drawing out the epidemic processes and causing them to resemble those observed in random and scale-free networks. In practice, this lead to an epidemic that spreads further and requires greater effort to contain. The pathogen’s probability of being transmitted from an infected node to a susceptible one (i.e. the overall pathogen infectivity, π), does not emerge as the primary driver of epidemic dynamics, but rather acts as a modulator of the effect of a vector’s long distance dispersal probability (δ).

An important caveat of these results is that they are subject to the assumptions of the SIR model, which we used here to represent a situation of relatively prompt detection and intervention. Such a model would be a poor choice to describe the Southern-Italian *Xylella* outbreak, which has seen significant latency between infection, and detection and removal of infected trees and neighboring ones (Abbott, 2017). Considering that trees are now no longer removed from a considerable portion of *Xylella*’s Italian range [the so-called ‘infected zone’ (European Commission et al., 2017)], and that *Xylella* can survive in local plants other than olives (EFSA (European Food Safety Authority), 2016), the disease could persist even if it killed all the olive trees. Hence, a Susceptible-Infected (SI) model could represent the current situation more realistically. The SI model assumes that an infected node/orchard remains infected for an indefinite amount of time, which might be now occurring in Italy’s infected zone (European Commission 2017). At the same time, the Italian *Xylella* outbreak has raised the level of awareness and vigilance across Europe, to the extent that the intensive monitoring activities conducted in Spain justify the choice for a SIR model for Andalusia.

Assessing the infectivity of emerging pathogens is very complex (Lloyd-Smith et al., 2015), yet our results indicate that, in the context of plant pests, the inevitable uncertainty is not critical for mitigation strategies. We namely find that, regardless of a pathogen’s infectivity, the long-distance movements of potential infective material, and hence measures that might reduce it, have an outsized effect on the epidemic dynamics compared to local intervention. Although it is true that assessing the probability of LDDs (the parameter δ in our simulations) might possibly be even harder than quantifying infectivity, our results suggest that the latter aspect might not be critical at least for management/decision making. Unfortunately, this conclusion is not good news, as it reflects the finding that unless the probability of LDDs is reduced to near-zero, epidemic dynamics can quickly become very volatile and the required control efforts escalate. Moreover, the conservative assumption might be that in most natural, unmanaged, situations “background” δ is significantly higher than zero, implying that unless infectivity of the plant pathogen is extremely low, interventions to greatly reduce LDD will be critical. In this context, the case of *Xylella* in Andalusia may be paradigmatic; if we assume that the infectivity of *Xylella* is extremely low, then the epidemic will never turn into a large outbreak, regardless of LDDs, reducing the need for disease management. However, the *Xylella* epidemic in Southern Italy teaches us that this is unlikely, since the pathogen has become widely diffused, suggesting that the infectivity of *Xylella* is in fact quite high, as also suggested by controlled experiments (Cornara et al., 2017). Under the pessimistic [but not unrealistic, given its first detection in April 2018 (Junta de Andalucía, 2018)] scenario of a *Xylella* epidemic in Andalusia, our findings highlight how LDDs will play a fundamental role in determining the dynamics of the outbreak, and the associated management opportunities.

There are various studies comparing different intervention strategies (see, for example, Thompson et al., 2018). Our results suggest that efforts to prevent LDD events should be considered a form of intervention by themselves, rather than merely a complement to management strategies that directly target hosts, pathogens, and vectors. EU-legislation around the management of the *Xylella* epidemic in Southern Italy calls for containing the pathogen, among others, by removing infected plants and their susceptible neighbors in some parts of the infected zone, and through increased surveillance in a ‘containment’ zone (European Commission 2017). Although the importance of preventing the uncontrolled movement of potentially dangerous plant material (and particularly of susceptible hosts) to and from the infected zone has also been highlighted, specific measures have not always been taken to control the movements of people, vehicles, or of goods not directly related to the disease (Regione Puglia, 2016). There are obvious practical reasons for this, with a region-wide control on such movements being clearly unfeasible. Yet, our study highlights how the impossibility of eliminating LDD might compromise the efficacy of other control measures.

Given the very high local abundance of *Xylella* insect vectors (Martelli et al., 2016), there is a great chance that moving people/goods/cars accidentally disperse infectious vectors between the infected and the susceptible areas. It is unclear to what extent this mechanism has contributed to the continued northward spread of *Xylella* in Southern Italy (European Commission et al., 2017), but our results do not allow us to dismiss it as an important contributing factor, and doing so might be a grave oversight. Despite the practical challenges, reducing the probability of LDD, far from being a complement to other management strategies, might be key to preventing or managing plant disease outbreaks, and may even be prioritized over other control strategies when resources are scarce. If achieved, constrained LDD events can substantially reduce the risk of outbreaks, by shaping infectious dynamics in a way that simplifies the implementation of quarantine. In practice, however, natural, stochastic agents of long-distance dispersal, such as strong winds for diseases with airborne vectors or pathogens, carry an inherent (and not negligible) risk for outbreaks. Such events can greatly set back eradication efforts, and may even see them abandoned altogether, as was the case when hurricane winds in 2004 spread Asiatic Citrus Canker (the bacterium *Xanthomonas axonopodis* pv. *citri*) over large parts of Florida (Gottwald and Irey, 2007).

In a broader picture, the geographic range of pathogens can be limited by their thermal tolerance rather than the availability of vectors (see Gutierrez et al., 2011), causing thermal tolerance to sometimes determine the extent of the epidemic network over which successful LDD events leading to infection may occur. Some research suggests this is the case for *X. fastidiosa*, which was first discovered in Europe in 2013 in the Salento peninsula of the Apulia region of southern Italy where infestations of the pathogen are currently found, and where an ongoing containment program is in progress (see Ponti et al., 2016). However, south-to-southeasterly Sirocco winds are a prominent climatic feature in this area (Ulbrich et al., 2012) and would greatly facilitate northward LDD of infective vectors of *Xylella* (Cornara et al., 2018, 2019) to northern non-infected areas of Apulia and beyond. However, without consideration of vector action, Bosso et al. (2016a,2016b) used field observations and the correlative ecological niche model Maxent (Phillips et al., 2006) to predict the prospective geographic distribution of the pathogen and found that it roughly coincides with the currently infected area of Salento. This result suggests that climatic factors might indeed limit the spread of *Xylella* independent of local and long distance vector dispersal (as we accounted for in our analysis by using the index of *Xylella* climatic suitability by Gutierrez et al., 2011).

In this work we have focused on monoculture landscapes (meant here in a broad sense as large areas dominated by a single crop), modelling the spread of a pathogen targeting a single host species. Yet, our overall conclusions can be virtually extended to systems with higher diversity of crops targeted by a more generalist pathogen, even though the additional complexity stemming, for example, from differences in pathogen’s virulence against different hosts would clearly require further modelling and analytical efforts (as it would be for the countless possible real world and simulated scenarios combining different degrees of host/crop diversity and pathogen host specificity).

Without taking those additional steps, and keeping the focus on monocultures, it is easy to identify various ecological mechanisms related to the links between pathogens’ infection dynamics and host density/availability that make it clear why a low diversity in agricultural landscape at the regional scale might encourage the spread and persistence of diseases (Anderson and May, 1979). Both the case of Citrus Canker in Florida and of *Xylella fastidiosa* in Southern Italy are notable examples where a single pathogen had benefitted from high host availability causing large environmental and economic losses. However, assuming, as a general rule, that low biodiversity and/or high density of suitable hosts increase the pathogen’s chances to succeed depicts an overly simplified scenario, which might well capture local scale processes depending on the chances of a pathogenic individual to find a suitable host, but not that much regional ones.

There, things get more complicated, revealing potential inconsistencies between the theoretical advantages provided by the spatial nature of the underlying networks depicting infection pathways, and the actual epidemic dynamics observed in several real world situations. Our work here reconciles theory with empirical observations, by showing how a small world effect triggered by extremely rare long distance dispersal can obliterate the structural robustness of agricultural spatial networks. On the up-side, embracing biodiversity in landscape planning might, in the long-term, help avoid plant health emergencies over which we currently have no more control than we have over hurricane winds.

## Data availability

The maps detailing the distribution of olive orchards in Andalusia are available from the “Land Use and Land Cover Information System of Spain (SIOSE)” at goo.gl/WyvHSJ. All the code and data needed to replicate the analyses can be obtained from https://github.com/giovannistrona/xylella.

## Contributions

G.S., P.S.A.B. and C.C. conceived the research; G.S., C.C., L.P. and A.P.G. designed and performed the analysis; G.S., S.F., C.C. and P.S.A.B. led the writing; all authors contributed to the final interpretation and writing of the manuscript.

## Declaration of Competing Interest

The authors declare no competing interests.
