# Supplementary material for: Small world in the real world: Long distance dispersal governs epidemic dynamics in agricultural landscapes
Source: Epidemics. 2020 Mar;30:100384. doi: 10.1016/j.epidem.2020.100384 (PMC7086151; doi:10.1016/j.epidem.2020.100384)
Supplement: Supplementary file 1 [file mmc1.docx]

**Supporting Information**

**Figure S1. Temporal trajectories of within node prevalence and node removal probability.** (**A**) The fraction of hosts (i.e. trees) infected within an infected node (i.e. orchard), *N_i_(t)*, increases through time (in months) as a Gompertz growth modelled by the equation $N_{i}\left( t \right)=exp\left[ -14.069\times exp\left( \frac{-0.25}{Months}\times t \right) \right]$. The probability of an infected node (e.g. orchards) to be “removed” from the network (for example, by clear-cutting, or pesticide treatment) (**B**) is also modelled by a Gompertz curve, but with a faster growth, according to the equation $r\left( t \right)=exp\left[ -14.069\times exp\left( \frac{-1}{Months}\times t \right) \right]$.

**Figure S2. Distribution of *rd* values used to model long distance dispersal events between two fields.** The role of *rd* values in our modelling procedure is illustrated in Equation 7 in the main text. *rd* values were sampled from a folded normal distribution with mean 50 km and standard deviation 150 km.

**Movie S1. Long distance connections drives epidemic processes.** In the animation, we provide a simple visual explanation of how the addition of random connections (10% of original number of edges) between nodes arranged in a regular lattice (with 900 nodes) can modify dramatically epidemic dynamics. The model used in the simulation is a SIR model with recovery probability equal to 1 (i.e. where infected nodes are removed from the network exactly one step after becoming infectious), with an infection infectivity of 0.6. Epidemic simulations are synchronized in the two panels. Throughout the simulations, we kept track, at each step, of the number of infected, removed, ‘quarantined’, and ‘saved’ nodes. The number of ‘quarantined’ nodes is the number of susceptible nodes neighboring infected nodes, and hence that should be ideally removed to halt the epidemic at the target step. Such number is tracked as a measure of the potential effort that would be required at the different stages of the outbreak to halt the epidemic, but quarantine is not actually implemented. The number of ‘saved’ nodes indicate the number of nodes that could be potentially secured by the application of quarantine (as defined above); ‘saved’ nodes at a given moment are therefore all the susceptible nodes in the network at that moment not neighboring infected nodes(and hence not target of quarantine).
