# Supplementary figures and images for: Small world in the real world: Long distance dispersal governs epidemic dynamics in agricultural landscapes
Source: Epidemics. 2020 Mar;30:100384. doi: 10.1016/j.epidem.2020.100384 (PMC7086151; doi:10.1016/j.epidem.2020.100384)

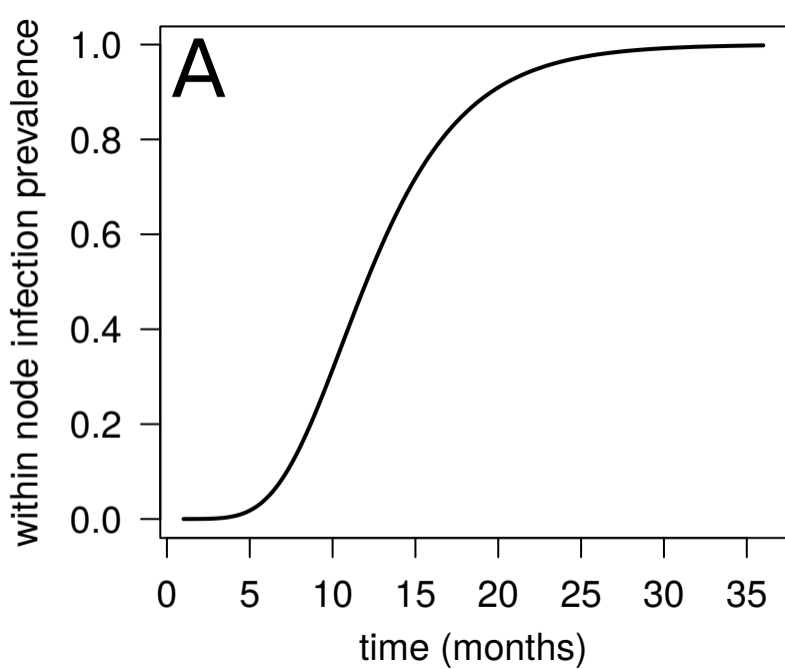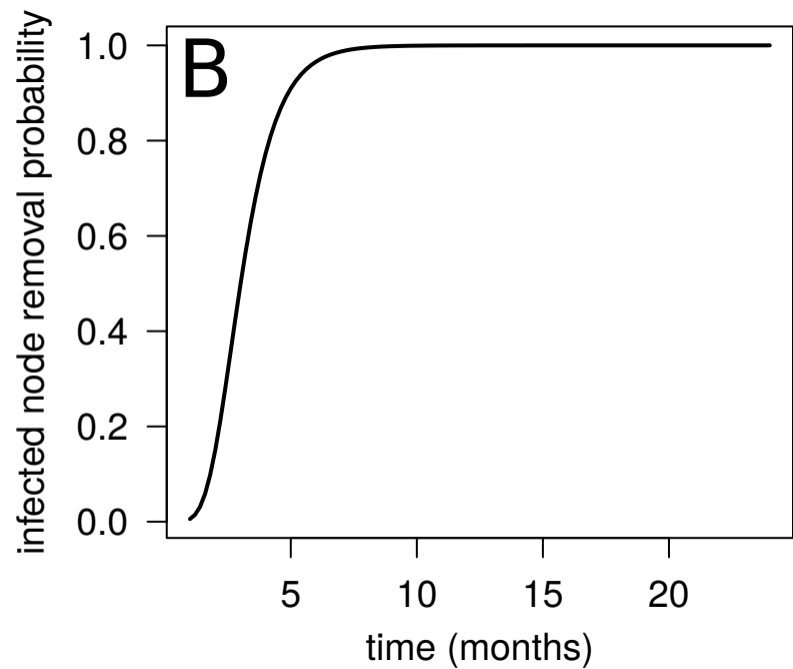

Supplement: Supplementary file 2 [file mmc2.pdf]

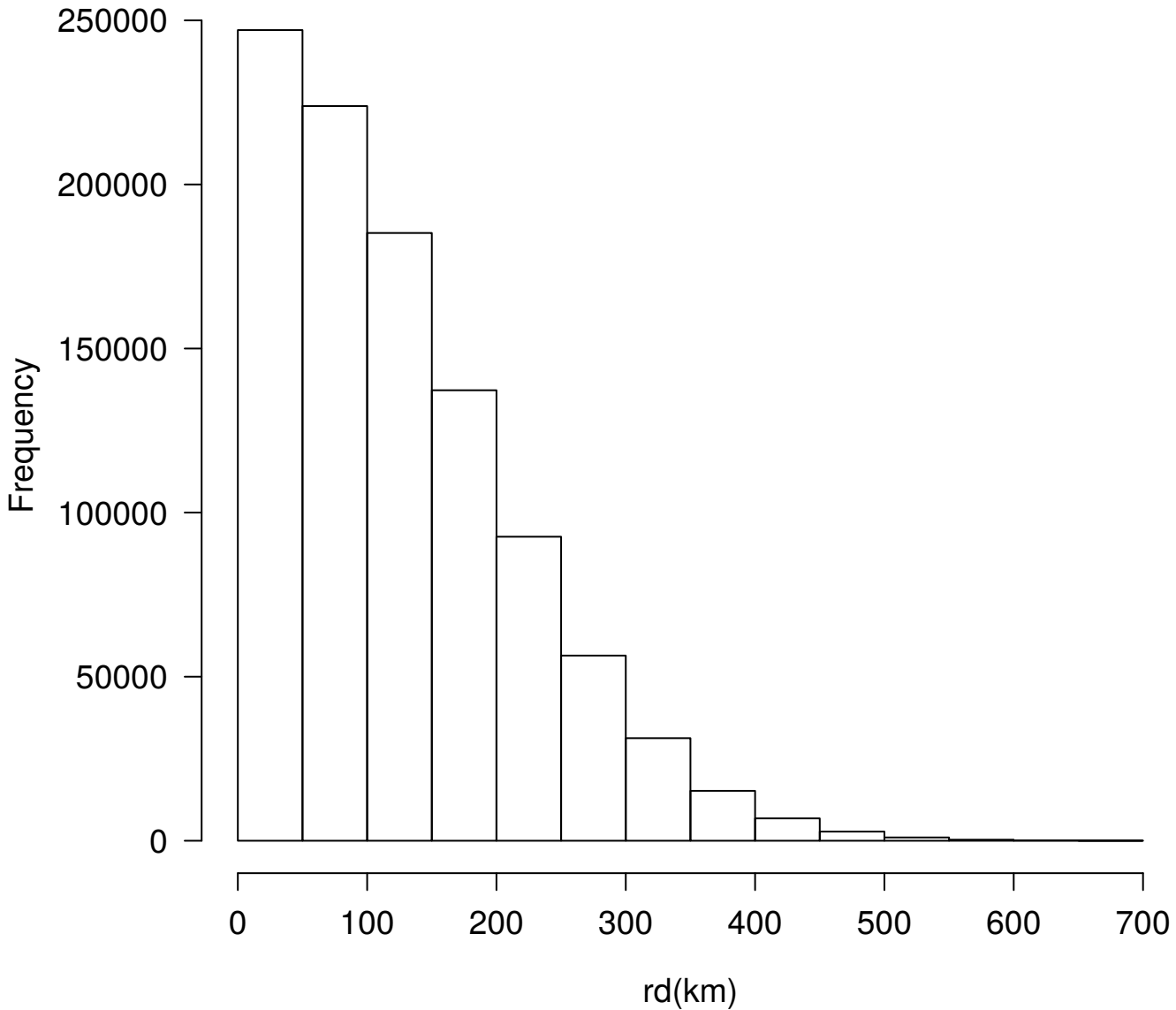

Supplement: Supplementary file 3 [file mmc3.pdf]
